# An individual-specific understanding of how synchrony becomes curative: study protocol

**DOI:** 10.1186/s12888-025-06539-3

**Published:** 2025-06-06

**Authors:** Sigal Zilcha-Mano, Yael Bouknik, Michal Malka, Tal Krasovsky

**Affiliations:** Department of Psychology, University of Haifa, Mount Carmel, Haifa, 31905 Israel

**Keywords:** Synchrony, Therapeutic relationship, Trait-like, State-like, Multi-modal, Mechanisms of change

## Abstract

**Background:**

Interpersonal interactions are a fundamental part of daily life, shaping mental health in profound ways. Yet, the mechanisms by which these interactions influence mental health remain poorly understood.

**Aims:**

This research is the first to systematically and prospectively test the conceptual framework proposed by Zilcha-Mano (2024), which introduces the concept of an individual-specific synchrony signature—a trait-like characteristic that distinguishes individuals based on their unique patterns of synchronizing across interpersonal relationships and contexts. It builds on the framework's challenge to the prevailing assumption that higher levels of synchrony are universally beneficial, instead positing that synchrony becomes curative when tailored corrections are made to an individual’s signature. Specifically, for synchrony to serve as a mechanism of therapeutic change, the direction and magnitude of the required synchrony adjustments must be tailored to align with the unique characteristics of the individual’s synchrony signature.

**Methods:**

This research uses psychotherapy as a case demonstration of curative relationships, offering a contained environment that combines the authenticity of real interpersonal dynamics with the sterile precision of laboratory conditions for examining synchrony dynamics. Study 1 uses the innovative Synchrony Interaction Paradigm to investigate the existence and characteristics of individual-specific synchrony signatures in a sample of 68 participants, including individuals with major depressive disorder (MDD; *N* = 34). Study 2 examines how tailored adjustments to these signatures facilitate therapeutic change, using a randomized controlled trial with 78 individuals diagnosed with MDD assigned to therapy targeting interpersonal mechanisms or to a waiting list. Both studies employ multimodal markers (e.g., motion, acoustic, physiological, hormones, and facial expressions) to disentangle the stable trait-like components of synchrony from state-like deviations occurring in real-time interactions with humans and virtual humans.

**Implications:**

This research redefines synchrony as an individual-specific mechanism of change, offering insights into its multi-modal nature and advancing a personalized framework for understanding its effect on mental health. The findings bridge critical gaps in synchrony research and contribute to more targeted and effective therapeutic interventions.

**Trial registration:**

clinicaltrials.gov Identifier: NCT06749392 submitted on December 12 st 2024. Trial status: Recruitment has not started yet

**Supplementary Information:**

The online version contains supplementary material available at 10.1186/s12888-025-06539-3.

Interpersonal synchrony, the spontaneous rhythmic and temporal coordination of reciprocal actions, emotions, and physiological processes between individuals, is a pervasive phenomenon integral to human interactions [1–3]. Synchrony has long been regarded as a key driver of collaborative, affiliative, and curative relationships [4, 5]. Its potential role in improving mental health through interpersonal relationships has sparked growing interest, particularly in psychotherapy [6]. However, the field is currently at a crossroads, with mixed empirical findings challenging the widespread theoretical assumption that “more synchrony is better.” [1, 4, 5, 7, 8] Building on the conceptual framework proposed by Zilcha-Mano [9], this research examines individual-specific synchrony signatures, shifting from generalized assumptions to tailored understanding and interventions. Using psychotherapy as a case demonstration, it empirically tests how synchrony can transform relationships into curative ones through individual-tailored changes in synchrony signatures.

## What is known: the state of the art

Synchrony is central to human interaction, beginning in infancy and persisting over the lifespan in relationships with caregivers [10], peers [11], romantic partners [12, 13], and even strangers [14]. Synchrony is perceived as playing an evolutionary role by fostering social cohesion and bonding, enabling humans to maintain larger and more interconnected social networks [15]. Advances in technology have expanded the ability to study synchrony across modalities, including motion [6, 16], physiology [2, 17], acoustics [18, 19], and facial expressions [20], allowing researchers to capture synchrony automatically at a relatively high resolution and low cost [21].

The rapid growth of research in the field has brought to light mixed results in studies on synchrony. While some research has found positive associations between synchrony and mental health outcomes, such as improved collaboration, and affiliative relationships [4, 5], other studies report null or even negative findings [4, 22, 23]. These inconsistencies arise, at least in part, from a “one-size-fits-all” approach [6, 24, 25], characterized by the assumption that “the higher the synchrony, the better”, which fails to account for individual differences and the contextual variability inherent in synchrony [26, 27]. Furthermore, most studies focus on within-modality synchrony, neglecting the potentially rich interplay between modalities (e.g., acoustic and motion synchrony), both within-individual (e.g., the individual’s acoustic and movement synchrony) and between-individuals (e.g., the individual’s acoustic and the partner’s movement synchrony), and the implications of these for understanding interpersonal dynamics [2, 28].

In psychotherapy, synchrony has been studied mainly in relation to therapeutic alliance and treatment outcomes [9, 21, 29, 30], particularly for depression. Although some findings suggest that higher levels of synchrony are associated with better mental health outcomes [6], others reveal no association or even detrimental effects [26]. For example, higher motion synchrony has been linked to poorer outcomes in certain subgroups, raising questions about the validity of generalized assumptions [27]. Similar patterns of mixed results have been observed not only in the relationship between synchrony and treatment outcomes but also in its association with the therapeutic alliance [27, 31–33]. This body of work underscores the need for a more precise understanding of synchrony that accounts for individual-specific patterns and their interplay with therapeutic processes and outcomes.

## The proposed theoretical conceptualization: an individual-specific trait-like synchrony signature

This research builds on and seeks to systematically and prospectively test the conceptual framework proposed by Zilcha-Mano [9], which shifts from the “the higher the better” theoretical assumption to a personalized understanding of synchrony. Central to this proposed framework is the concept of an *individual-specific trait-like synchrony signature*, an individual-specific pattern of synchrony that manifests across interactions and reflects the characterizing mode of activation of the synchrony system. Zilcha-Mano [9] hypothesized these signatures to fall into three main types: normative, hyperactivating, and deactivating. Each type represents distinct patterns of going in and out of sync, balancing social attunement and autonomy. The normative synchrony signature is characterized by dynamic oscillations between synchrony and desynchrony, reflecting a healthy balance between interpersonal connection and individual autonomy. By contrast, hyperactivation represents an exaggerated tendency to fall in sync, potentially blurring self-other boundaries, while deactivation reflects avoidance of synchronized interactions, prioritizing autonomy at the expense of interpersonal connection [34]. These individual differences in synchrony signatures are proposed here to be pivotal in understanding the potential of relationships to become curative, as well as their broader impact on social and mental health outcomes [9].

A key innovation of this framework is the distinction between *trait-like* and *state-like* components of synchrony, where the trait-like component represents an individual’s stable, cross-contextual synchrony signature and the state-like component captures momentary deviations from it [9]. Understanding the interplay between these components is essential to determining when, for whom, and how synchrony can serve as a curative mechanism. For example, individuals with a deactivation signature may benefit from interventions that increase synchrony levels, whereas those with hyperactivation may require strategies to reduce them. By tailoring interventions to the individuals’ synchrony signature, this framework aims to optimize therapeutic processes and outcomes. This theoretical model builds on insights from psychotherapy research on mechanisms of change, particularly the distinction between trait-like and state-like components [35–39]. It posits that synchrony signatures can serve as transdiagnostic mechanisms underlying mental health and interpersonal functioning, offering a new lens through which to study and enhance the curative potential of relationships.

## The present research

The overarching goal of this research is to investigate how, for whom, and under what conditions interpersonal synchrony can serve as a mechanism underlying the curative potential of interpersonal relationships. This study builds on the conceptual framework proposed by Zilcha-Mano [9], which is based on two foundational premises: (a) each individual possesses a trait-like synchrony signature that characterizes them across interpersonal interactions, and (b) inducing individually tailored changes in a maladaptive synchrony signature can transform a relationship into a curative one.

This research leverages psychotherapy as a unique context for investigating interpersonal synchrony. By encapsulating authentic curative interactions within the structured framework of its sessions, psychotherapy provides an ideal setting for systematically examining the role of synchrony as an individual-specific mechanism of therapeutic change. The study focuses on psychotherapy for major depressive disorder (MDD) as a case demonstration. As the leading cause of disability worldwide [40, 41], MDD represents a critical global health challenge. Moreover, most research on synchrony has been conducted with individuals diagnosed with MDD [16, 17, 42–44], providing a robust foundation for this investigation. By focusing on MDD, this research builds on existing knowledge to deepen our understanding of how interpersonal synchrony can transform relationships into curative ones, offering innovative solutions to a pressing mental health issue.

This research has four primary aims: (a) establish the existence and characteristics of individual-specific synchrony signatures across various modalities and interactions; (b) examine how these signatures vary across individuals and contexts, as well as their potential to drive curative processes in psychotherapy; (c) develop a multimodal framework integrating within- and between-modality synchrony to provide a comprehensive understanding of interpersonal dynamics; and (d) leverage these insights to design personalized interventions, facilitating the development of targeted strategies to improve interpersonal relationships and mental health. This work aims to lay the foundation for a new field of personalized synchrony research, with transformative implications for psychotherapy and broader applications in improving interpersonal relationships over the lifespan.

The present research employs the *Synchrony Interaction Paradigm (SIP*, [45]), a novel experimental procedure involving multiple dyadic interactions with humans and virtual humans, developed by us to explore the existence and characteristics of individual-specific synchrony signatures (Study 1). It will also examine whether, for whom, and under what conditions these signatures are stable versus amenable to change, and how deviations from them contribute to mental health improvements (Study 2). Together, these studies are intended to establish synchrony as an individual-specific mechanism of change, advancing personalized mental health interventions and offering novel insights into the mechanisms of curative interpersonal processes.

Specific aims:

Aim 1: Map the individual-specific interpersonal synchrony system by disentangling the trait-like synchrony signature from state-like context-specific and dyad-specific deviations. The study will also investigate associations between the synchrony signature, other trait-like characteristics of the individual, and mental health outcomes (Study 1).

Aim 2: Determine how deterministic the trait-like synchrony signature is by identifying for whom and when changes are anticipated. Specifically, the study will examine whether the synchrony signature transfers to the relationships with the therapist, whether and how it changes throughout treatment, and whether such potential changes are associated with improvements in mental health (Study 2).

By addressing these aims, this research aims to redefine the role of synchrony in mental health, offering a systematic and personalized framework for therapeutic interventions that optimize treatment outcomes and enhance the quality of interpersonal relationships. This approach leverages the unique characteristics of individuals with diverse trait-like profiles, ensuring interventions are tailored to meet the specific needs of each person.

## Method

### Study 1

#### Design

Study 1 investigated the synchrony system by examining its constituent elements, normative operation, individual differences, and associations with mental health. To achieve these objectives, we will use the Synchrony Interaction Paradigm (SIP [45]). As part of the SIP framework, participants will engage in multiple dyadic interactions with both humans and virtual humans (strangers in a virtual reality environment), the latter simulated to exhibit hyperactivation or deactivation of the synchrony system. The SIP was designed to enable the collection of synchrony data across various modalities, time points, and interactions, facilitating the identification of an individual-specific trait-like synchrony signature. Additionally, data on factors potentially contributing to the trait-like synchrony signature, state-like deviations from it, and participants' mental health will be systematically assessed to provide a comprehensive understanding of the synchrony system.

#### Participants

A total of 68 participants (34 with MDD) will be recruited through advertisements, offering NIS 480 to compensate for their time and effort. The sample size was chosen to provide adequate power to test our hypotheses (see power analysis below).

##### Individuals without MDD

A total of 34 participants without MDD will participate. *Inclusion criteria.* (a) scores under 14 on the 17-item Hamilton Rating Scale for Depression (HRSD [46]) at two consecutive assessments, one week apart; (b) age between 18 and 65 years; (c) Hebrew language proficiency; (d) provision of written informed consent. *Exclusion criteria.* (a) Current or past psychiatric disorders including substance use disorders and the use of any psychotropic medications; (b) current MDD based on the International Neuropsychiatric Interview, (MINI [47, 48]; Structured Clinical Interview for DSM5); (c) history of organic mental disease; (d) current risk of suicide or self-harm (HRSD [46] suicide item > 2); (e) currently in psychotherapy.

##### Individuals with MDD

A total of 34 participants with MDD will participate. *Inclusion criteria.* (a) current MDD based on the International Neuropsychiatric Interview (MINI [47, 48]; Structured Clinical Interview for DSM5) and scores above 14 on the 17-item HRSD at two consecutive assessments, one week apart; (b) for participants using psychiatric medication, the dosage must be stable for at least three months before the beginning of the study, and they will be asked to maintain stable dosage during the treatment; (c) age between 18 and 65 years; (d) Hebrew language proficiency; (e) provision of written informed consent. *Exclusion criteria.* (a) current or past schizophrenia or psychosis, bipolar disorder, or severe eating disorder, demanding close medical monitoring; (b) history of organic mental disease; (c) current risk of suicide or self-harm (HRSD [46] suicide item > 2); (d) current substance abuse disorder; (e) being currently in psychotherapy. After completing Study 1, participants with MDD who meet the inclusion criteria for Study 2 will be given the choice to participate in Study 2.

##### **Procedure**^1^(see Figs. 1 and 2).

**Fig. 1:**
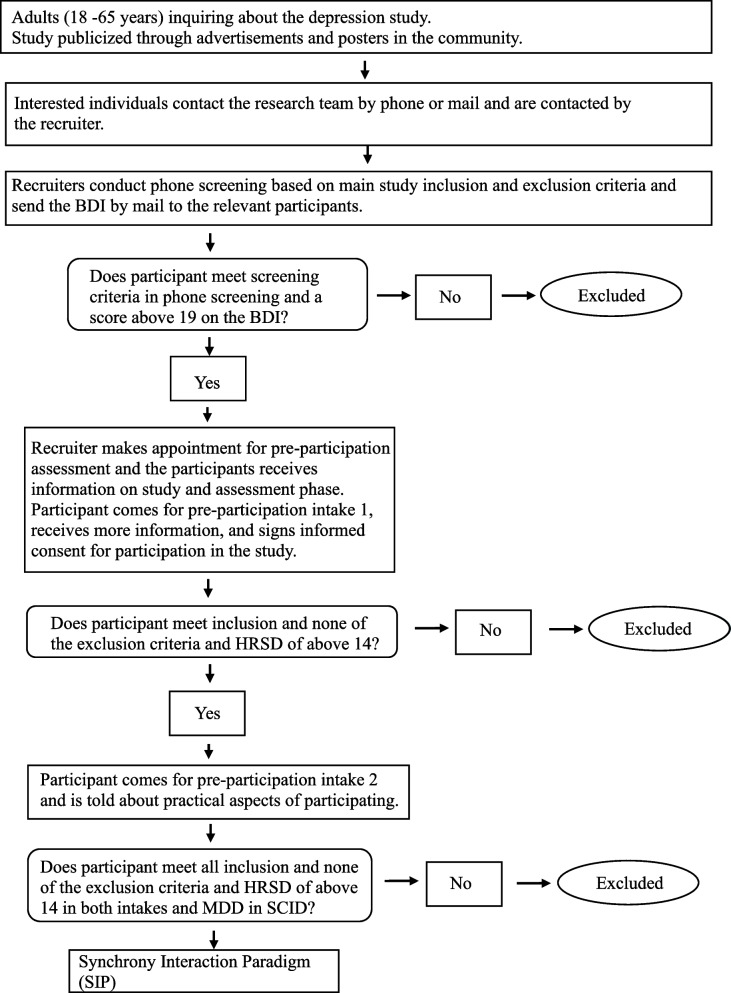
Study 1-Depression population flowchart

**Fig. 2:**
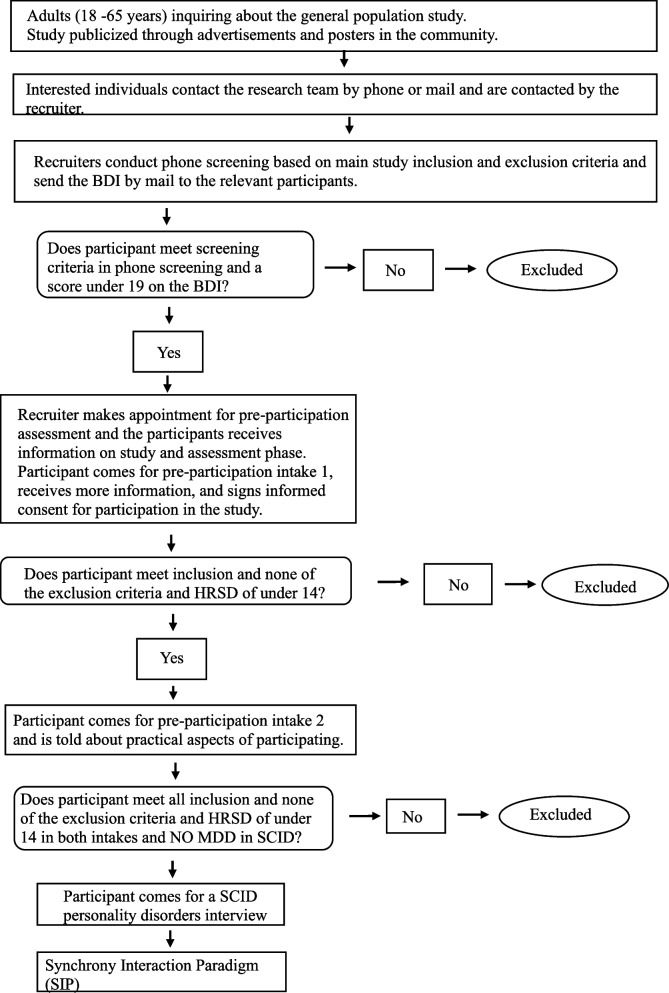
Study 1-General population flowchart

##### Recruitment

Recruitment will be through self-referral, based on advertisements in local media, and online publications (social media, etc.). Interested individuals will be instructed to apply by email or by phone.

##### Screening

In an initial phone screening, members of the research team will inform the applicants about the study details and procedure and the inclusion and exclusion criteria. To evaluate the severity of depressive symptoms, applicants who meet the eligibility criteria and consent to participate in the research intake procedure will be asked to complete the Beck Depression Inventory (BDI-II [49]).

##### Assessment meetings

**Informed consent.** Participants will be contacted to schedule an initial intake meeting, during which the researchers will provide detailed written and oral information about the procedure of the planned study and encourage applicants to ask questions. The researchers will inform applicants that all interactions will be videotaped and that they have the right to withdraw from the research at any time. The researchers will also inform applicants about the full research procedure and assessments, its safety, and possible risks.

**Depression assessment.** To measure the presence and severity of baseline MDD symptoms, an HRSD [46] interview will be conducted by trained interviewers. At the second assessment meeting, the HRSD [46] will be administered again. In addition, the MINI [47, 48] will be administered to measure the presence and severity of baseline MDD symptoms and comorbid conditions.

**Personality disorder assessment.** Following the second interview, a third face-to-face meeting will be scheduled, in which a different interviewer will assess comorbid personality disorders using the Structured Clinical Interview for DSM- IV Personality Disorders (SIDP [50].

**Baseline measures.** A first face-to-face assessment meeting will be scheduled only for applicants who agree to participate and sign the informed consent form. At the first assessment meeting, participants will be asked to complete a battery of questionnaires that includes demographic information, concurrent medication, comorbid conditions, several personality and interpersonal characteristics, and baseline symptom self-reports.

The clinical interviews (HRSD [46], the MINI [47, 48], SIDP [50] and Self-Understanding of Interpersonal Patterns Scale (SUIP [51], see below) will be administered by independent, rigorously trained research interviewers. After completing two months of extensive training and successfully achieving high reliability, the interviewers will observe another interviewer at work. Only then will they begin to administer the interviews, first with a trained interviewer observing them in the first several weeks, and later by themselves. During the trial period, the reliability of the interviewers will be evaluated weekly.

##### The SIP paradigm

**Preparation phase for the SIP.** Participants who meet the eligibility criteria and consent to participate in the study will proceed to the SIP [45]. Prior to their lab visit, participants will be asked to identify and describe six recent relational interactions they perceive as problematic, involving significant others. These interactions will be documented using the protocol of the SUIP ([51], see details below).

**The synchrony interaction paradigm.** During the SIP, participants will share one of the six problematic interactions identified during the preparation phase to each of six distinct dyadic partners: an adult family member (age above 18), a best friend, a romantic partner (or another close friend or family member for those without a romantic partner), a previously unmet individual, and two virtual humans. The six interactions will be randomly assigned to the dyadic partners. In the interaction with the virtual human, participants will recount a problematic interaction while seated in the same room in which they met their human listeners. Using a virtual reality (VR) platform, participants will see a virtual representation of the room and interact with the virtual human. Each virtual human corresponds to a real person physically present in the room (stranger). Participants will view an avatar representation of themselves, which mirrors their real-time movements. The movements of the virtual human are manipulated in real-time using software to either hyperactivate or deactivate synchrony with the participant's movements.

Each interaction will be shared in a 20-min dyadic session, during which the participant will recount their problematic interaction in detail. Listeners (both in the real world and VR) will be instructed to demonstrate empathy, listen attentively, and ask clarifying questions when appropriate, to ensure the interaction is as ecologically valid as possible. Participants will first engage with the real word listeners in a randomized sequence. Following these interactions, they will engage with virtual humans, also presented in a random order. This setup ensures a comprehensive evaluation of synchrony across both human and virtual interactions.

### Measures

#### Treatment outcomes: primary and secondary

##### Primary outcome

Severity of depressive symptoms, measured using the HRSD [46], will be the primary outcome measure of the trial. The HRSD is a 17-item measure administered by clinical interviewers. Depressive symptom severity is calculated by summing up all 17 items.

##### Secondary outcomes

Secondary outcomes will be assessed using a set of self-reported measures (i.e., Beck Depression Inventory [49]; Beck Anxiety Inventory [52], Outcome Questionnaire [53]; quality of life (Quality of Life Enjoyment and Satisfaction [54]); In addition, insight measures will be assessed using the SUIP interview [51].

##### Measures predicting trait-like synchrony signature

**Interpersonal measures.** Anxiety and avoidance attachment orientations (i.e., Experience in Close Relationships [55], and difficulties in interpersonal relationships (i.e., Interpersonal Problems Inventory [56]).

**Intrapersonal measures.** Emotion regulation (Emotion Regulation Questionnaire [57]; Difficulties in Emotion Regulation Scale [58]); self-esteem (i.e., Rosenberg Self-Esteem Scale [59]); personality traits (Five Factor Personality Inventory [60]); empathy (Interpersonal Reactivity Index [61]); mentalization (i.e., Certainty About Mental States Questionnaire [62]); exposure to trauma events (Posttraumatic Diagnostic Scale for DSM-5 [63]), and insight (SUIP [51]).

##### Measures predicting state-like deviations from the trait-like signature

**Measures characterizing each dyadic relationship will be administered for each partner of the interaction**. Tendency and efficacy of intrinsic interpersonal emotion regulation (i.e., Interpersonal Regulation Questionnaire [64]) and relationship closeness (i.e., experiences in close relationships—relationship structures [65]).

**Measures characterizing each dyadic relationship in the context of the specific interaction will be administered for each partner of the interaction before and after each interaction.** Affect (Positive and Negative Affect Scale [66]); the expectation from interpersonal relationship and realization (i.e., Personal Report of Interpersonal Relationships [67]; personalized power relations [68]), and the working alliance (Working Alliance Inventory, WAI [69]).

##### Measures to assess multi-modal synchrony

Synchrony will be measured based on four modalities (Table 1).

**Table 1:** The 28 candidate markers of synchrony from 4 modalities

Dimensions	Features to be used	Details	Data acquisition	Data quantification
Physiological	Heart rate variability (GSR +) [70]	HRV may indicate mental and biological functioning. [70] EDA is a measure of the activity of the sympathetic nervous system, which is related to a state of increased arousal [71]. Their potential importance in measuring synchrony has been demonstrated [24, 72, 73]	Shimmer 3	Python EDA analysis [72]
	Electrodermal activity[71]			
Motional	Two predefined motion regions of interest (ROIs): (a) head region (b) gesture and body region [33, 74]	Motion energy is defined as the difference in pixel values between consecutive video frames. The detection of frame-by-frame changes enables quantification of the changes in predefined ROIs. Its potential importance in measuring synchrony has been demonstrated [6, 16]	Video	Motion energy analysis [75]
	Cross-correlation analysis of amplitude and quality of movement across multiple body segments		Video; AR51 motion capture; Unreal engine (Epic Games, Inc.)	
Acoustic	Fundamental frequency, speech rate, pause, jitter	Vocal acoustics was found to convey information regarding the arousal [76, 77] and the emotional [78–80] and interpersonal [81, 82] characteristics of individuals Its potential importance in measuring synchrony has been demonstrated. [83, 84]	Audio	Praat software package [85]
Facial expressions	20 Action units that interact to generate different facial expressions. [86]	AFFDEX classifier identifies basic emotions using a combination of 20 anatomically-based facial actions. Its potential importance in measuring synchrony has been demonstrated. [30, 87]	Audio	iMotions Biometric Research Platform, open face

**Interaction with virtual humans.** Interactions with virtual humans will take place in a VR environment in the same room in which participants met their human listeners. The participant will wear an immersive VR headset and will be part of a virtual environment designed to mimic a real room. Participants will view their own movements mirrored in the virtual environment and hear the real voice of the listener (a stranger). However, the listener’s movements in the virtual environment will not reflect their actual movements but rather pre-recorded gestures will be used to manipulate the synchrony between the virtual human listener and the participant, either by hyperactivating or deactivating it. To do so, we will create a “bank” of listening gestures based on previous listening data during dyadic interactions. The 3D movements of the participant will be recorded, characterized, and used in real time to manipulate the movements of the virtual human listener. Specifically, hyperactivation of the synchrony system will be operationalized by triggering gestures that will be time- and amplitude-locked to the participant’s movements, and deactivation of the system will be operationalized by triggering gestures not synchronized to those of the participant.

In the VR environment, a head-mounted display (HMD) (Meta Quest 3) will provide a computer-generated image of the virtual room, similar in all aspects (size, furniture, etc.) to the actual one in which the participants interacted with the other human figures. A markerless motion capture system (AR51) will provide full-body movement kinematic data in the virtual environment, allowing the participant to be represented by a first-person perspective virtual body whose virtual movements reflect the real body movements of the participant in real time. The result is a sensorimotor congruence (because of visual and proprioceptive alignment) that creates an illusion of ownership and agency of the participants over their virtual bodies. After participants are engaged in dyadic interactions with multiple other humans, they will enter the virtual reality lab and will be fitted with a Meta Quest 3 HMD. They will be familiarized with their virtual bodies seen from a first-person perspective and seated on a real chair, conforming to a virtual chair. Shortly thereafter, a virtual human listener will enter the room.^2^

#### The VR environment

The Meta Quest 3 has a nominal field of view of 110°, with a resolution of 2,064 × 2,208px per eye displayed at 72 Hz. The experiment will take place in the same room where all interactions with humans will occur. The virtual environment will be implemented using the Unreal engine (Epic Games, Inc.). Participants’ virtual body skeletons will be rendered using a proprietary SDK by AR51 from Unreal. One male and one female virtual human character will be selected from an existing bank and will be used for all male and female participants. Approximate character age will be selected to fall within the normal range of the sample population of participants, and their looks will be casual.

#### Overview of statistical analyses

For each participant in interaction with each interacting-other, we will collect 28 features from the four types of modalities. We will compute the autoregressive (“counting on the self”) and interactive (“counting on the other”) effects for each of the 28 features of the participant with each of the 28 features of the interacting other to assess cross-modality interactions. Similarly to previous research from our lab, we will calculate the autoregressive and interactive effects using a series of Actor-Partner Interdependence Models (APIM[88]), which estimate cross-lagged associations between the participant and each interacting partner, measuring within-session synchrony. We will use the autoregressive (actor) effect, also referred to as *intra*personal effect, to estimate “counting on the self,” which will serve to describe the deactivation pattern. We will use the interactive (partner) effect, referred to as *inter*personal effect, to estimate “counting on the other,” which will serve to describe the hyperactivation pattern [89]. Data will be detrended before analysis. To calculate actor and partner effects for each individual, we will include random effects on the participant and interacting-other actor and partner effects and intercept. We will extract these random effects for each participant and interacting-other in each dyad using empirical Bayes residuals.

##### Identifying the individual-specific trait-like signature

We will blend all autoregressive and interactive effects of all possible pairs of the 28 features of the participant and the 28 features of the interacting-other to explore whether clusters of single individuals emerge from the observations. We will use Support Vector Machine (SVMs) to conduct two types of classification tasks: participant and relationship. Our lab has expertise in using SVMs to identify patterns in high-resolution data [90]. This method is based on the relatively straightforward idea that, given two classes of data graphed onto a two-dimensional space based on some identifiable features, a theoretically infinite number of lines can be drawn to divide the data. SVM has been used to identify the best dividing line as one that is maximally distant from the nearest data point of each class, providing the largest possible buffer space between the two classes. This buffer space defines the optimal separating hyperplane (OSH). The OSH separates the classes of data and minimizes the risk that new data will be incorrectly classified [91]. SVM is not limited to two- or even three-dimensional space, nor to linearly-separable classes. Using the SVM, we will be able to explore whether homogenous individual-specific signatures incorporating all the interactions of a given individual will emerge and whether they will be different from all the interactions in which the given individual did not take part (i.e., which will form the other individuals’ signatures).

##### Identifying individual differences in the individual-specific signature

To explore the existence of the proposed three patterns of individual differences, we will use the most differentiating features of autoregressive and interactive effects, as found in the SVM analyses described above. We will use the autoregressive (actor) effect to estimate the deactivation pattern and the interactive (partner) effect to estimate the hyperactivation pattern. To explore whether the participants’ and interacting-others’ actor and partner effects create three between-clients clusters (normative, hyperactivating, deactivating), we will use an unsupervised machine learning algorithm, based on the k-means clustering method, developed by Hartigan and Wong [92].We chose the K-means clustering method because of its many advantages, including the fact that it is not model-based and applies optimization algorithms to define participants’ assignment to clusters [93, 94].We will implement the k-means clustering on the participants’ autoregressive and interactive effects and use the average silhouette method to determine the optimal number of clusters that best fit the data [95],computing the average silhouette of observations for different values of k. We will choose the number of clusters, k, as the one that maximizes the average silhouette over a range of possible values for k. We will use the R function *kmeans* in the *stat* package for clustering.

We will characterize the clusters found and identify the features that best differentiate between them in two steps: (a) compare the clusters on demographic and clinical characteristics (using ANOVA) and (b) build a classification tree using the *party* R package to predict cluster membership. We will explore whether the above measures of the intrapersonal and interpersonal characteristics of the individual are associated with the synchrony signature, determining whether the individual-specific signature is associated with constructs related to needs for closeness/dependence vs. autonomy. To achieve this, we will conduct a *machine learning classification tree analysis,* using random forest variable selection and Monte Carlo simulation for multiple testing adjustments [96]. To assess the robustness and consistency of the clustering, we will cross-validate the procedure described above using the validation set approach. In this method, the dataset is divided randomly into training and validation sets. The number of clusters is determined by the silhouette. Next, we will identify the determined number of k-means clusters for each training sample, and build classification trees for that number of clusters. The training sample is based on *P* = 20%, 30%, …, 90% of all clients. We will use the data of the other clients (P—100%) as a validation sample to classify them into the new clusters based on the newly created classification tree. We have successfully conducted these procedures in previous projects [97]^.^

##### The association between individual differences in the signature and mental health

We will test whether individual differences in the signatures are associated with individual differences in mental health. We will use ANOVA to explore whether the clusters identified differ in levels of mental health (symptom reduction, mental health) to test our hypothesis that the normative pattern will be associated with better mental health than either the deactivation or the hyperactivation pattern.

#### Power analysis

The main analyses will focus on the main outcome measure (HRSD [46]) and mapping the individual-specific signature and the association with mental health.

The sample size for Study 1 was chosen to provide adequate power to test our hypotheses. To determine the required sample size, we simulated data based on 1568 variables (784 combinations of the 28 features × 2, for the autoregressive + interactive features) for 60 participants with 6 repeated measurements, assuming a normal distribution with a variance of 1 for each variable, using cross-validation with 6 folds. In each fold, one measurement of each participant was removed and the classifier was used to classify the removed observation. In 1000 simulations, we obtained 83% correct classification.

##### Identifying individual differences in the activation of the synchrony system

Based on the power calculations and equations provided by Dalmaijer et al., [98] ‏we expect around 35–45 features to emerge in the support vector machine (SVM) (within- and between-modalities, both autoregressive and interactive features). We expect to find three equally sized subgroups. Between these subgroups, we expect large size differences (the first features in the SVM; Cohen’s d = 0.8) in 15–20, and medium size differences (the next features in the SVM; Cohen’s d = 0.5) in 20–25 features. Thus, based on the equation, the total expected separation is Δ = 3.8–4.3 and a power of 80% is expected for *N* = 60. Given attrition and missing data, we will sample 68 individuals: half non-depressed individuals and half with depression.

## Study 2

### Design

Study 2 investigates how deterministic the trait-like synchrony signature (as mapped in study 1) is in the absence of intervention, and whether changes in the signature are associated with changes in mental health as a result of intervention. The target relationships will be those with the therapist. All participants in Study 2 will have undergone the process described in Study 1 before starting their participation in Study 2. First, participants will be enrolled in a first session with a therapist. Second, participants will be allocated to a randomized control trial (RCT) and randomized to either a 16 weeks waiting list or supportive-expressive treatment (SET [99, 100]) with the therapist they met at the first session. To ensure balance across age, sex, family status, baseline 17-item HRSD [46], and personality disorders, a minimization algorithm will be used. At the end of treatment, the participants will again undertake the SIP [45]) vis-a-vis the same figures they interacted with before the therapy. At the end of the trial, participants who were randomized to the waiting list will be enrolled in treatment. The trial will follow the Standard Protocol Items: Recommendations for Interventional Trials (SPIRIT) checklist [101] requirements for an RCT [101] with a design that includes both treatment and waiting list conditions.

### Participants

**Fig. 3:**
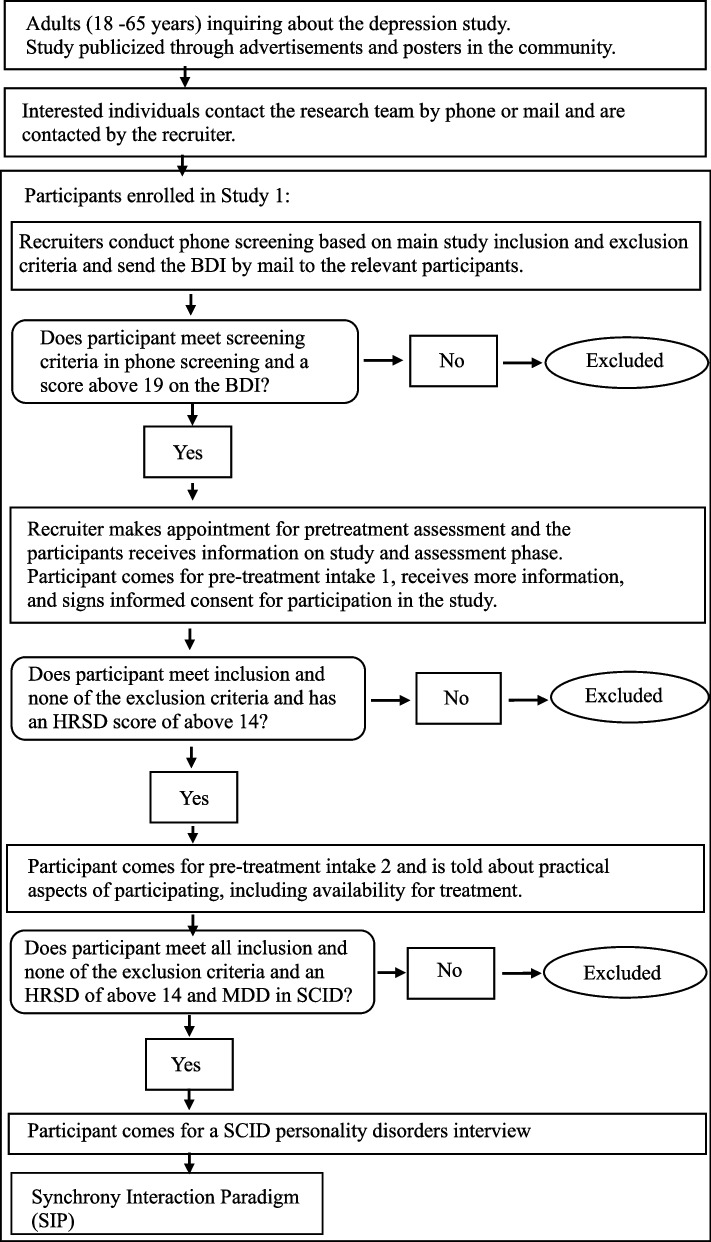
Study 2 Flowchart

Participants will be 78 patients diagnosed with MDD. Recruitment will take place through advertisements, offering free treatment at the University of Haifa Psychotherapy Research Lab clinic (Fig. 3). All participants will have previously enrolled in Study 1. The sample size was chosen to provide adequate power to test our hypotheses.

#### Inclusion criteria

(a) current MDD based on the MINI (Structured Clinical Interview for DSM5 [47, 48], and scores above 14 on the 17-item HRSD [46] at two consecutive assessments, one week apart; (b) for participants using psychiatric medication, the dosage must be stable for at least three months before the beginning of the study, and they will be asked to maintain stable dosage during the treatment; (c) age between 18 and 65 years [102]; (d) Hebrew language proficiency; (e) provision of written informed consent.

#### Exclusion criteria

(a) current or past schizophrenia or psychosis, bipolar disorder, or severe eating disorder, demanding close medical monitoring; (b) history of organic mental disease; (c) current risk of suicide or self-harm (HRSD [46] suicide item > 2); (d) current substance abuse disorder; (e) being currently in psychotherapy.

### Treatments

Patients will be enrolled either in a treatment of 16 50-min sessions or in a waiting list. The treatment will be SET, which is manualized time-limited psychodynamic therapy adapted for depression [99, 100]. The treatment includes the use of supportive techniques, such as affirmation and empathic validation, as well as more expressive techniques, such as interpretation, confrontation, and clarification (SET [99, 100]). Participants who were enrolled in the waiting list will receive treatment 16 weeks later.

### Therapists

The treatment will be provided by experienced therapists. Before starting to treat patients, therapists will receive intensive training on the SET manual [99, 100] and will treat a pilot case coded for adherence to ensure adequate levels of proficiency. Therapists will participate in weekly group supervision during the duration of the trial, making extensive use of videotaped sessions for feedback. An international expert in SET with more than 25 years of experience will be involved in and will accompany the process of supervision.

### Fidelity check

To ensure that therapists adhere to the manual and are competent in the technique, they will attend weekly supervisions in which videotaped sessions will be viewed to identify and analyze instances of deviation from the manual. In addition, each therapy session will be videotaped, and external evaluators will rate the therapists' adherence and competence. Adherence and competence in treatment will be evaluated using the Penn Adherence-Competence Scale (PACS [103]). PACS consists of three subscales: supportive component, expressive component, and general therapeutic behaviors. The raters will be supervised by an international expert on the use of the PACS, with vast experience in using the PACS in RCTs on SET. In cases of low adherence or insufficient competence, the therapist and the supervisor will be informed and relevant supervision tools will be deployed (e.g., [104, 105](.

#### Procedure and randomization (see Fig. 4)

**Fig. 4:**
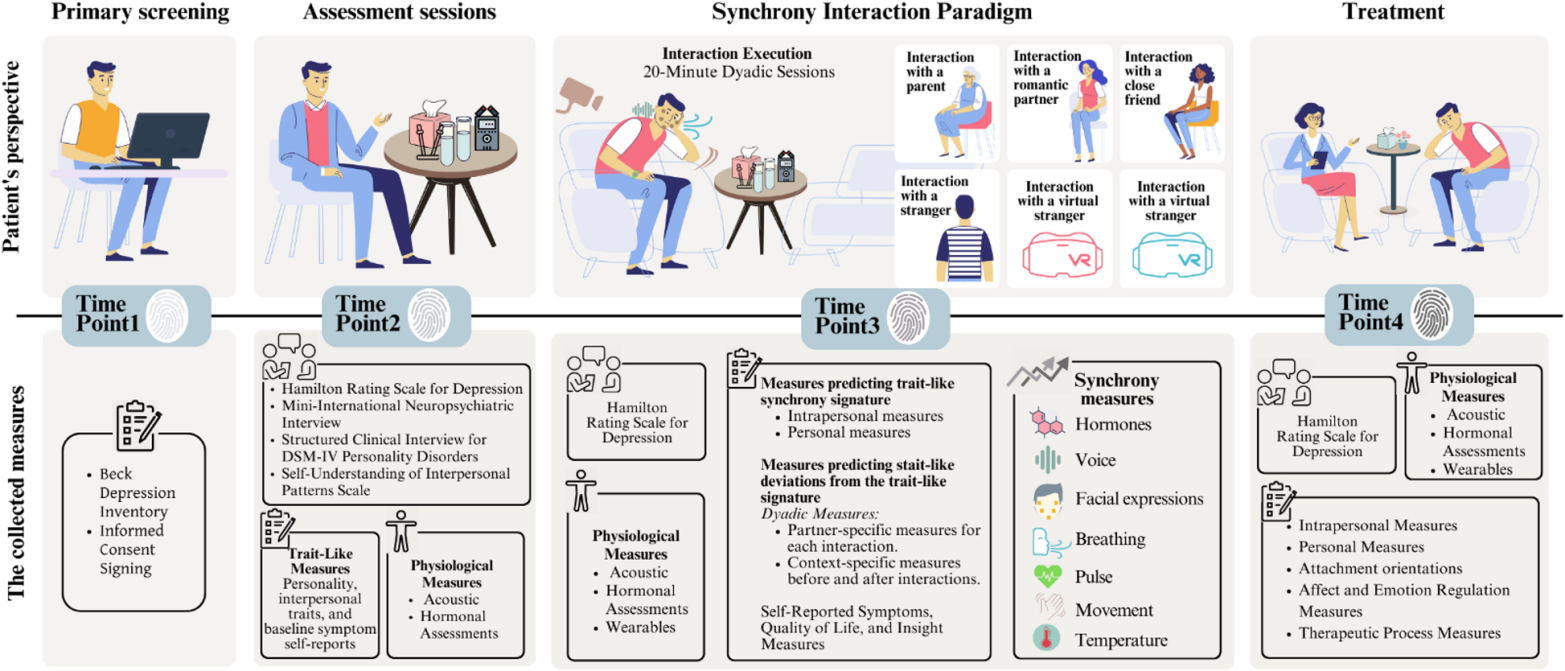
Study timeline: key assessment points and data measures of Study 2

##### Recruitment and pre-study phase

Recruitment will take place through advertisements offering free treatment. All participants meeting inclusion and exclusion criteria for Study 2 will first be enrolled in Study 1 to identify their synchrony signature and then be enrolled in Study 2 (see details above in the procedure section for Study 1).

##### Randomization

Patients who meet the eligibility criteria and agree to participate in the study will be randomly assigned to either treatment of 16 50-min sessions or to a waiting list. Randomization will be performed by an independent institution, not involved in the study. Applicants who have not met the inclusion criteria or have met the exclusion criteria and therefore are not eligible for the study, but request psychotherapy, will be referred to alternative care. All outcome evaluator interviews will be videotaped, and 30% of the tapes will be randomly assigned and rerated by independent raters.

##### Treatment phase

**Process and outcome measures.** During the active phase of treatment, the HRSD [46] and self-report questionnaires will be administered weekly. Participating patients will be asked to complete self-report measures assessing their symptoms (e.g., BDI-II[49]) before each session and self-report measures assessing treatment processes following each session. Levels of insight will be measured at baseline and at the end of the treatment (at week 16). Every fourth session during the course of treatment, patients will be asked to complete computerized tasks and to provide saliva samples. In addition, the MINI [47, 48] and SIDP [50] will be measured at weeks 8 and 16.

**Termination**. Patients who choose to withdraw from treatment before the end of the active treatment phase will receive a dropout questionnaire. Their therapists will also be asked to complete a dropout questionnaire [106].

#### Synchrony Interaction Paradigm (SIP)

After completion of the active phase of the treatment (16 weeks), the participants will undertake the SIPSynchrony Interaction Paradig [45] vis-à-vis the same figures they interacted with before the therapy, adhering to the protocol detailed in Study 1.

##### Follow-up

Assessments will be scheduled at 3, 6, 12, and 24 months after the end of the active phase. The follow-up assessment sessions will include HRSD [46], MINI [47, 48], and a battery of self-report questionnaires. At 6 and 12 months, the SIDP [50] will also be administered. To avoid bias, patients will be asked not to join other psychotherapeutic programs and not to change medication dosages.

### Measures

#### Treatment outcome

##### Main outcome

Severity of depressive symptoms, measured using the HRSD [46], will be the primary outcome measure of the trial. The HRSD is a 17-item measure administered by clinical interviewers. Depressive symptom severity is calculated by summing up all 17 items.

##### Secondary outcomes

**Symptoms measures.** Self-reported symptoms (i.e., Beck Depression Inventory [49]; Beck Anxiety Inventory [52]), Outcome Questionnaire [53], and quality of life (Quality of Life Enjoyment and Satisfaction [54]).

**Interpersonal measures**. Anxiety and avoidance attachment orientations (i.e., Experience in Close Relationships [55], and difficulties in interpersonal relationships (i.e., Interpersonal Problems Inventory [56]).

##### Therapeutic process measures

Working alliance (WAI [69]), therapeutic interventions (Multi-theoretical List of Therapeutic Interventions, MULTI [107]), psychological flexibility (Psychological Flexibility Questionnaire [108], expectation from treatment (Outcomes and Experiences Questionnaire [109], and insight measures will be assessed using the SUIP [51].

#### Overview of statistical analyses

Analyses will be conducted using multilevel nested models of observations nested within clients nested within therapists to account for the hierarchical structure of the data. All analyses will be conducted using the Proc Mixed procedure in SAS. All have been used extensively in our lab. To test whether in the absence of intervention, the trait-like signature is deterministic, we will test the percentage of variance explained in synchrony with the therapist from the trait-like signature identified using the procedure developed in our lab (see Study 1). To determine whether changes occur in the synchrony signature in the treatment vs. the control condition, we will use a model of time by condition (treatment vs. control conditions) predicting synchrony development over the course of treatment. To determine whether transformation in the trait-like synchrony signature is associated with strengthening of the alliance and improvement in mental health in the treatment condition, we will use two models of synchrony by condition (treatment vs. control conditions) predicting alliance and mental health, respectively, over the course of treatment. To test whether those with poorer trait-like synchrony benefit most from deviations toward a healthier pattern, we will test the between-levels interaction between the trait-like signature and changes in it by treatment condition (treatment vs. control conditions) in predicting changes in mental health.

#### Power analysis

The main analyses will focus on the main outcome measure (HRSD [46]).

To calculate the required sample size for the moderation models (based on the most complex model), we used an approach modeled on Monte Carlo simulations, estimated using the R code generated by the MLPowSim software package, applying 10,000 Monte Carlo simulations, which produces more accurate results for power estimates than other methods for relatively small sample sizes. The power calculation was based on effect sizes from our pilot data. Assuming alpha = 0.05 and 16 repeated measurements of the outcome variable (including missing data), the simulations indicated a required sample size of 78 participants to ensure a power of at least 0.80.

#### Ethical considerations for Study 1 and Study 2.

The design, procedure, and informed consent form of the studies were approved by the ethics committee of the University of Haifa (approval number: 048/24, Date: February 14, 2024). Any modifications of the protocol will be reported to the committee. As detailed above, participants will be given comprehensive information about the study procedure both orally and in writing. The information describes the possible implications of their participation, including potential risks, inconvenience, and benefits. In the event of a significant adverse incident (e.g., severe suicide risk), the RISK protocol will be activated. Specifically, a special committee including three licensed clinical psychologists trained to deal with such situations will immediately take charge.

#### Data management for Study 1 and Study 2

To ensure the confidentiality of the data at all stages of the research process, a detailed data management plan is deployed. A data monitoring committee will supervise data collection. An identification number will be assigned to each participant on enrollment and will be used for data registration. The correspondence list will be kept securely in a password-protected computer. After completion of the trial, the raw data will be kept in a locked drawer in a locked room at the University of Haifa Psychotherapy Lab. After the dataset is de-identified and anonymized, researchers who will be approved by the steering committee will be able to access the dataset.

#### Dissemination policy for Study 1 and Study 2

Study results will be disseminated to the public at academic conferences and in peer-reviewed journals.

## Discussion

Despite profuse investments in seeking to improve the understanding of how interpersonal relationships become curative, our knowledge remains limited [4, 22]. This study builds on the framework proposed by Zilcha-Mano [9], which shifts from the conventional “the higher the better” [1] theoretical premise to an individual-specific perspective. By emphasizing the role of personal synchrony signatures [9], this research tests the framework's challenge to existing paradigms and seeks to establish a personalized understanding of synchrony. This innovative framework has the potential to fundamentally alter our understanding of how synchrony serves as a potential mechanism by which interpersonal relationships influence mental health.

At the core of this research are transformative assumptions: the existence of a personal synchrony signature, its categorization into three main types (normative, deactivating, and hyperactivating), and the hypothesis that curative changes in synchrony must be tailored to an individual’s unique signature [9]. These yet to be tested but promising assumptions redefine how synchrony is conceptualized and applied across disciplines. By generating a comprehensive understanding of the synchrony system—its normative operation and individual differences in activation—this study offers novel insights into when and for whom changes in synchrony are most effective. Specifically, it explores how synchrony signatures are generalized to new interactions or modified under certain conditions, using the therapeutic relationship in psychotherapy targeting interpersonal change as a proof of concept.

This research has extensive potential influence. Tailoring synchrony to individual-specific signatures represents a paradigm shift from a one-size-fits-all approach to personalized interventions [36, 110, 111]. The personalized framework could revolutionize mental health care by facilitating the development of targeted strategies that enhance treatment outcomes for diverse populations, including parent–child relationships, romantic partnerships, and peer interactions [112]. Moreover, the implications extend beyond mental health, offering tools to understand and improve interpersonal dynamics in various societal and cultural contexts [9].

A key methodological innovation of this study is the further development of the SIP[45], a multimodal dyadic paradigm, capable of assessing and extracting the individual-specific synchrony signature in real time. This technological advancement sets the stage for real-time interventions to enhance synchrony, improve mental health outcomes, and lay the groundwork for large-scale monitoring and individualized treatment strategies. Additionally, the study bridges the gap between trait-like and state-like mechanisms in psychotherapy [9, 25, 36, 37, 113] and synchrony research [16, 38], testing for the first time the existence of personal synchrony signatures as mechanisms of change.

Funded by the European Union, this protocol is part of a larger, ambitious project aimed at transforming our understanding of interpersonal synchrony and its role in curative relationships. Studies 1 and 2 serve as the foundation for Study 3, which will manipulate synchrony to achieve therapeutic outcomes. By shifting from a generalized framework to an individual-specific perspective, this research offers a groundbreaking approach to both research and clinical practice, paving the way for a new era in personalized mental health care and the study of interpersonal relationships.

## Supplementary Information

Supplementary Material 1.

## Data Availability

No datasets were generated or analysed during the current study.

## Abbreviations

MDD: Major depressive disorder
SIP: Synchrony interaction paradigm
HRSD: Hamilton rating scale for depression
MINI: Mini-international neuropsychiatric interview
BDI: Beck depression inventory
SIDP: Structured clinical interview for DSM- IV personality disorders
SUIP: Self-understanding of interpersonal patterns scale
VR: Virtual reality
WAI: Working alliance inventory
GSR: Galvanic skin response
HRV: Heart rate variability
EDA: Electrodermal activity
ROIs: Regions of interest
HMD: Head-mounted display
APIM: Actor-partner interdependence models
SVMs: Support vector machine
OSH: Optimal separating hyperplane
RCT: Randomized control trial
SET: Supportive-expressive treatment
SPIRIT: Standard protocol items: recommendations for interventional trials
PACS: Penn adherence-competence scale
MULTI: Multi-theoretical list of therapeutic interventions

## Footnotes

1 In Study 1 and Study 2, we will use validated versions of all questionnaires in Hebrew.

2 In the event of a delay in establishing the VR environment, a small subset of participants will engage in sessions with real, non-virtual human listeners outside the VR environment.
